# Implementation of an early rule-out pathway for myocardial infarction using a high-sensitivity cardiac troponin T assay

**DOI:** 10.1136/openhrt-2021-001769

**Published:** 2021-11-25

**Authors:** Dennis Sandeman, Maaz B J Syed, Dorien M Kimenai, Kuan Ken Lee, Atul Anand, Shruti S Joshi, Lorraine Dinnel, Philip R Wenham, Ken Campbell, Mary Jarvie, Donna Galloway, Mhairi Anderson, Bappa Roy, Jack P M Andrews, Fiona E Strachan, Amy V Ferry, Andrew R Chapman, Sarah Elsby, Mark Francis, Robert Cargill, Anoop S V Shah, Nicholas L Mills

**Affiliations:** 1 Cardiology Department, NHS Fife, Kirkcaldy, UK; 2 Division of Clinical and Surgical Sciences, University of Edinburgh, Edinburgh, UK; 3 British Heart Foundation Centre for Cardiovascular Science, University of Edinburgh, Edinburgh, UK; 4 Biochemistry Department, NHS Fife, Kirkcaldy, UK; 5 Emergency Department, NHS Fife, Kirkcaldy, UK; 6 Information Services, NHS Fife, Dunfermline, UK; 7 London School of Hygiene & Tropical Medicine, London, UK; 8 Usher Institute, University of Edinburgh, Edinburgh, UK

**Keywords:** acute coronary syndrome, biomarkers, chest pain

## Abstract

**Objectives:**

Patients with suspected acute coronary syndrome and high-sensitivity cardiac troponin (hs-cTn) concentrations below the limit of detection at presentation are low risk. We aim to determine whether implementing this approach facilitates the safe early discharge of patients.

**Methods:**

In a prospective single-centre cohort study, consecutive patients with suspected acute coronary syndrome were included before (standard care) and after (intervention) implementation of an early rule-out pathway. During standard care, myocardial infarction was ruled out if hs-cTnT concentrations were <99th centile (14 ng/L) at presentation and at 6–12 hours after symptom onset. In the intervention, patients were ruled out if hs-cTnT concentrations were <5 ng/L at presentation and symptoms present for ≥3 hours or were ≥5 ng/L and unchanged within the reference range at 3 hours. We compared duration of stay (efficacy) and all-cause death at 1 year (safety) before and after implementation.

**Results:**

We included 10 315 consecutive patients (64±16 years, 46% women) with 6642 (64%) and 3673 (36%) in the standard care and intervention groups, respectively. Duration of stay was reduced from 534 (IQR, 220–2279) to 390 (IQR, 218–1910) min (p<0.001) after implementation. At 1 year, all-cause death occurred in 10.9% (721 of 6642) and 10.4% (381 of 3673) of patients in the standard care group (referent) and intervention group, respectively (adjusted OR 1.02, 95% CI 0.88 to 1.18).

**Conclusion:**

In patients with suspected acute coronary syndrome, implementing an early rule-out pathway using hs-cTnT concentrations <5 ng/L at presentation reduced the duration of stay in hospital without compromising safety.

**Key questions**

**What is already known about this subject?**

- Cardiac troponin using high-sensitivity assays can accurately risk stratify patients with suspected acute coronary syndrome.
- The use of early rule-out pathways using high-sensitivity cardiac troponin (hs-cTn) has been recommended by European Society of Cardiology for patients with suspected myocardial infarction.

**What does this study add?**

- We show that the implementation of an early rule-out pathway, using an hs-cTnT assay, reduces duration of stay in hospital without compromising safety in a large, unselected and consecutive cohort of over 10 000 patients presenting with suspected acute coronary syndrome.

**How might this impact on clinical practice?**

- In patients with suspected acute coronary syndrome, implementing an early rule-out pathway, using an hs-cTnT concentration of <5 ng/L at presentation for risk stratification, reduced the duration of stay in hospital without compromising safety.
- The adoption of this approach in clinical practice could have major benefits for patients and healthcare providers.

## Introduction

Chest pain is a common presentation and is responsible for approximately 700 000 hospital admissions per annum in the UK alone.^1^ Given that the majority of patients with chest pain do not have acute myocardial infarction, strategies that safely identify low-risk patients in the emergency department and reduce the need for hospital admission would be of major benefit to both patients and healthcare providers.

High-sensitivity cardiac troponin (hs-cTn) is the gold standard biomarker for diagnosis of myocardial infarction.^2^ The Fourth Universal Definition of Myocardial Infarction requires a rise and/or fall in cardiac troponin concentrations above the 99th percentile to confirm the diagnosis.^2^ Previous studies have demonstrated that troponin concentrations below the limit of detection have a high negative predictive value and sensitivity to rule out myocardial infarction^3–7^ and these patients remain at low risk.^5 8–11^ Improved sensitivity of cardiac troponin assays has therefore enabled the development of multiple early rule-out pathways.^11–15^ Most studies evaluating early rule-out pathways have been observational in nature, combining the limit of detection with electrocardiographic criteria or risk scores.^6 16^ While a single measurement at presentation is more cost-effective than delayed testing,^5^ the clinical effectiveness and safety of this approach in practice remains uncertain.^17^

We have evaluated the efficacy and safety of implementing an early rule-out pathway using an hs-cTnT concentration <5 ng/L at presentation to risk stratify patients with suspected acute coronary syndrome.

## Methods

### Study design and participants

In this prospective single centre, controlled before and after study, we included consecutive patients with suspected acute coronary syndrome presenting to a secondary care hospital, the Victoria Hospital in Kirkcaldy, Scotland, between 25 November 2014 and 12 June 2017, in whom the attending clinician measured hs-cTnT. The hs-cTnT assay was introduced in our hospital on 25 November 2014. An early rule-out pathway using an hs-cTnT concentration of <5 ng/L at presentation to rule out myocardial infarction was introduced on 13 June 2016. Patients were excluded if they were not residents in Scotland, had a previous presentation during the study period or had an ST-segment elevation myocardial infarction as these patients were taken directly to a regional cardiac centre for primary percutaneous coronary intervention. All patients who had an hs-cTnT measurement on presentation to hospital since the introduction of the assay were included in the study.

Data collection and record linkage were performed with permission from the National Health Service (NHS) Caldicott Guardian. Individual patient consent was not sought as all data were routinely collected from the electronic patient record with no additional research procedures. Data were prospectively collected, de-identified, linked within a secure NHS safe haven. This study was conducted in accordance with the Declaration of Helsinki.

### Patient and public involvement

No patients were involved in setting the research question or the endpoint measures, nor were they involved in developing plans for design of the study. No patients were asked to advise on interpretation or writing up of results.

### Cardiac troponin assay and pathways

Throughout the study, hs-cTnT was measured on the Cobas e602 platform (Roche Diagnostics, Basal, Switzerland) which has a manufacture-specified limit of detection of 3 ng/L on this platform and a non-sex-specific 99th percentile upper reference limit of 14 ng/L.^18^

**Figure 1:**
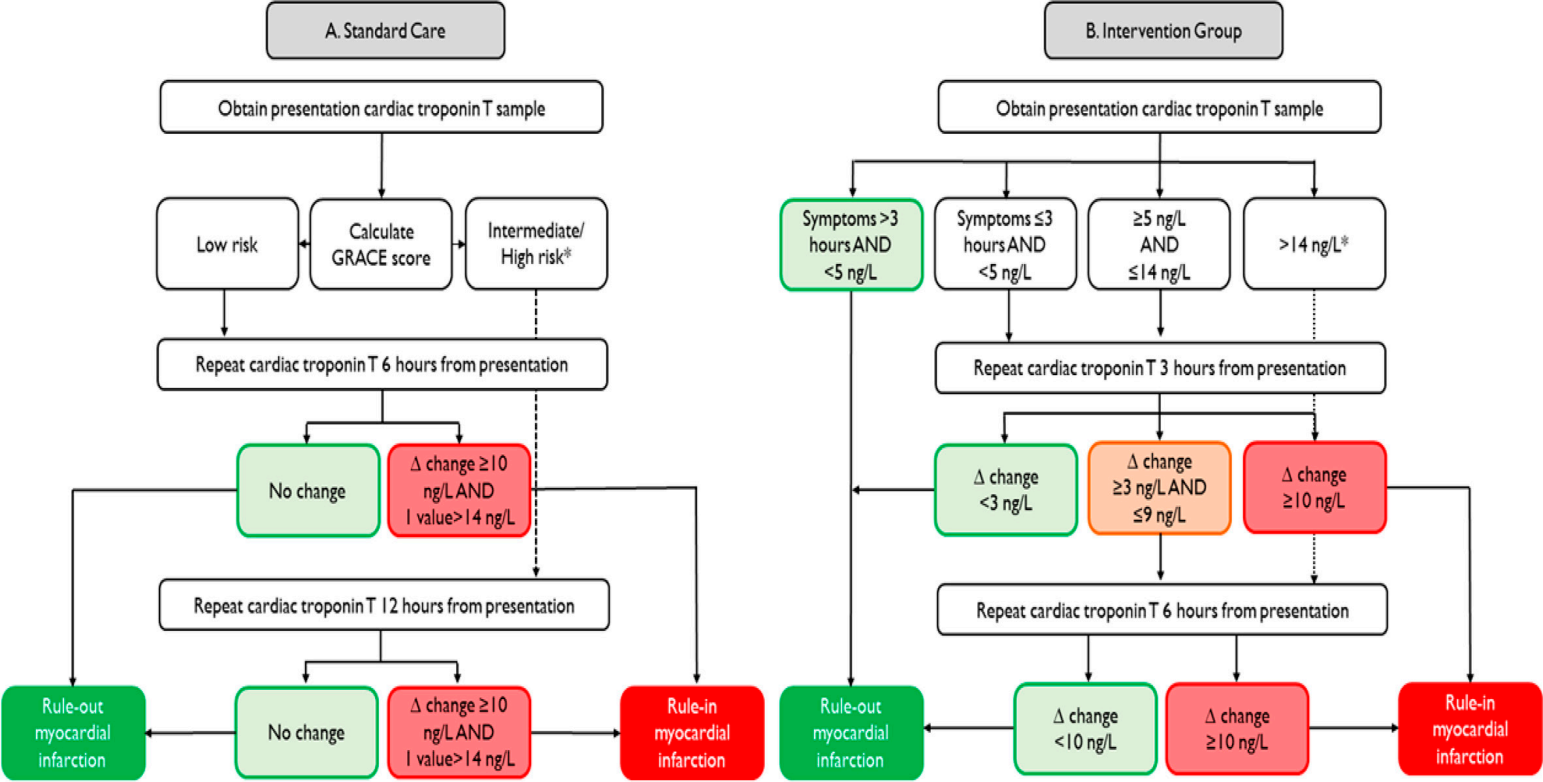
Standard care and intervention pathways flow chart showing the pathway used in the standard care and intervention group. Low risk in standard pathway was <1% death at 6 months and moderate to high risk was ≥1% of death at 6 months.

Patients with suspected acute coronary syndrome were included before (standard care) and after (intervention) implementation of the early rule-out pathway. During standard care, patients had troponin measured at presentation and a repeat measurement at 6 or 12 hours from symptom onset in those considered low (<1% death at 6 months) and high risk (≥1% death at 6 months) according to the GRACE score (figure 1).^19^ During the implementation period, patients with undetectable hs-cTnT concentrations (<5 ng/L) and symptoms >3 hours at presentation were identified as low risk and considered for immediate discharge. In patients presenting within 3 hours of symptom onset or those with hs-cTnT concentrations within the reference range from 5 to 14 ng/L, testing was repeated 3 hours from presentation and those without a significant change (<3 ng/L at 3 hours) were identified as low risk and considered for early discharge.^20^

During both phases, myocardial infarction was considered in patients with an hs-cTnT concentration >14 ng/L and an absolute change of ≥10 ng/L following repeat testing based on previous research with this assay.^21^ In the 3 months prior to implementation of the early rule-out pathway, regular educational presentations, as well as written and online materials, were provided to clinical staff. While clinical staff were encouraged to follow the early rule-out pathway, the final diagnosis and management during the entire study period were at the discretion of the treating clinician.

### Efficacy and safety endpoints

The primary efficacy endpoint was duration of hospital stay. The secondary efficacy endpoint was the proportion of patients discharged from hospital at 4 hours. The primary safety endpoint was all-cause death at 1 year. Secondary safety endpoints were all-cause death at 30 days and cardiovascular death at 30 days and 1 year. Information on cause of death was obtained using the NHS Central Register as previously described.^22^ Cardiovascular death was classified using the 10th revision of the International Classification of Diseases (ICD-10), and defined with ICD-10 codes (myocardial infarction=I21, I22; cerebrovascular disease=I60, I61, I63–66; heart failure=I110, I130, I1426, I50).

### Statistical analysis

We compared standard care and intervention in all patients with suspected acute coronary syndrome and stratified by hs-cTnT concentrations at presentation (<5 ng/L, between 5 and 14 ng/L, and >14 ng/L) (figure 1). For comparisons, we used the Student’s t-test and Mann-Whitney U test for normally and non-normally distributed continuous variables, respectively. Χ^2^ test was used for categorical variables. To evaluate the primary efficacy endpoint, we used linear regression to compare the duration of stay (natural log) between standard care and the intervention (reference=standard care), adjusted for age, sex, creatinine, diabetes mellitus, and prior myocardial infarction, heart failure or cerebrovascular disease. The primary and secondary safety endpoints were compared between the standard care and intervention groups using logistic regression in unadjusted (model 1) and then adjusted models for age, sex, renal function and comorbidity. Sample size calculations were performed using the duration of hospital stay as the primary endpoint. As duration of stay is non-normally distributed, a sample size calculation was done based on the approach by O’Keeffe *et al*.^23^ A sample size of 2503 patients in each group would give 90% power at an alpha of 0.05 for detecting a 20% difference in median duration of stay, where the duration of stay in the standard care group was 530 min. Statistical analyses were carried out using R V.3.6.3 (R Foundation for Statistical Computing, Vienna, Austria).

### Data availability statement

This study makes use of multiple routine electronic healthcare data sources that are linked, de-identified and held in our safe haven, which are accessible by approved individuals who have undertaken the necessary governance training. Summary data can be made available upon request to the corresponding author.

## Results

### Clinical characteristics of study population

We included 10 315 consecutive patients (54% men, mean age 64 years) with suspected acute coronary syndrome (table 1). Of these, 6642 (64%) patients were in the standard care group and 3673 (36%) patients were in the intervention group (figure 1). Mean age and sex were similar in both groups. The intervention group was less likely to have a history of myocardial infarction (7.6% vs 4.2%) or heart failure (4.7% vs 3.2%). In the standard care group (6642), the number and proportion of patients with hs-cTnT concentration <5 ng/L, between 5 and 14 ng/L, and >14 ng/L were 2188 (32.9%), 1885 (28.4%), and 2569 (38.7%), respectively. In the intervention group (3673), the number and proportion of patients with hs-cTnT <5 ng/L, between 5 and 14 ng/L, and >14 ng/L were 945 (25.7%), 1380 (37.6%), and 1348 (36.7%), respectively.

**Table 1:** Baseline clinical characteristics, efficacy and safety of patients with suspected acute coronary syndrome before and after implementation of early rule-out pathway

	Both groups	Standard care group				Intervention group			
		All patients	<5 ng/L	5–14 ng/L	>14 ng/L	All patients	<5 ng/L	5–14 ng/L	>14 ng/L
	(n=10 315)	(n=6642)	(n=2188)	(n=1885)	(n=2569)	(n=3673)	(n=945)	(n=1380)	(n=1348)
Age (mean)	63.57 (16.4)	63.77 (16.3)	50.31 (12.6)	65.25 (12.8)	74.14 (13.1)	63.20 (16.4)	48.04 (12.1)	63.02 (13.4)	74.01 (13.1)
Sex (male), n (%)	5571 (54.0%)	3628 (54.6%)	994 (45.4%)	1121 (59.5%)	1513 (58.9%)	1943 (52.9%)	368 (38.9%)	804 (58.3%)	771 (57.2%)
Medical history									
Myocardial infarction, n (%)	660 (6.4%)	507 (7.6%)	70 (3.2%)	151 (8.0%)	286 (11.1%)	153 (4.2%)	12 (1.3%)	64 (4.6%)	77 (5.7%)
Diabetes mellitus, n (%)	1823 (17.7%)	1177 (17.7%)	154 (7.0%)	345 (18.3%)	678 (26.4%)	646 (17.6%)	70 (7.4%)	218 (15.8%)	358 (26.6%)
Heart failure, n (%)	432 (4.2%)	315 (4.7%)	9 (0.4%)	36 (1.9%)	270 (10.5%)	117 (3.2%)	2 (0.2%)	16 (1.2%)	99 (7.3%)
Medication at presentation									
Antiplatelets, n (%)	3387 (32.8%)	2311 (34.8%)	346 (15.8%)	718 (38.1%)	1247 (48.5%)	1076 (29.3%)	86 (9.1%)	439 (31.8%)	551 (40.9%)
ACE inhibitor or ARB, n (%)	3192 (30.9%)	2095 (31.5%)	376 (17.2%)	692 (36.7%)	1027 (40.0%)	1097 (29.9%)	127 (13.4%)	484 (35.1%)	486 (36.1%)
Beta-blocker, n (%)	2723 (26.4%)	1810 (27.3%)	330 (15.1%)	560 (29.7%)	920 (35.8%)	913 (24.9%)	124 (13.1%)	355 (25.7%)	434 (32.2%)
Lipid-lowering therapy, n (%)	3931 (38.1%)	2623 (39.5%)	442 (20.2%)	854 (45.3%)	1327 (51.7%)	1308 (35.6%)	138 (14.6%)	549 (39.8%)	621 (46.1%)
Diuretic therapy, n (%)	1504 (14.6%)	1018 (15.3%)	59 (2.7%)	181 (9.6%)	778 (30.3%)	486 (13.2%)	22 (2.3%)	111 (8.0%)	353 (26.2%)
Laboratory results									
Creatinine (µmol/L) (SD)	93 (53)	94 (53)	75 (15)	85 (22)	116 (76)	92 (55)	73 (18)	83 (25)	113 (81)
Efficacy									
Duration of stay (minutes)	469 (220–2148)	534 (220–2279)	236 (180–484)	421 (215–1214)	2259 (665–6054)	390 (218–1190)	219 (170–329)	311 (215–838)	2567 (679–6191)
Four-hour discharge (yes)*	3431 (33.5%)	2150 (32.4%)	1149 (52.5%)	661 (35.1%)	340 (13.2%)	1281 (34.9%)	604 (63.9%)	512 (37.1%)	165 (12.2%)
All-cause death (%)									
At 1 year	1102 (10.7%)	721 (10.9%)	17 (0.8%)	73 (3.9%)	631 (24.6%)	381 (10.4%)	4 (0.4%)	48 (3.5%)	329 (24.4%)
At 30 days	386 (3.7%)	245 (3.7%)	1 (0.0%)	14 (0.7%)	230 (9.0%)	141 (3.8%)	1 (0.1%)	12 (0.9%)	128 (9.5%)
Cardiovascular death									
At 1 year	568 (5.5%)	364 (5.5%)	4 (0.2%)	20 (1.1%)	340 (13.3%)	204 (5.6%)	1 (0.1%)	17 (1.2%)	186 (13.8%)
At 30 days	221 (2.1%)	139 (2.1%)	1 (0.0%)	4 (0.2%)	134 (5.2%)	82 (2.2%)	1 (0.1%)	8 (0.6%)	73 (5.4%)

Continuous variables are presented as mean (SD) or median (IQR). Categorical variables are presented as n (%).

*Sixty-eight patients with missing data (45 patients from standard group and 23 patients from intervention group).

ARB, angiotensin receptor blocker.

### Efficacy of implementing the early rule-out pathway

**Figure 2:**
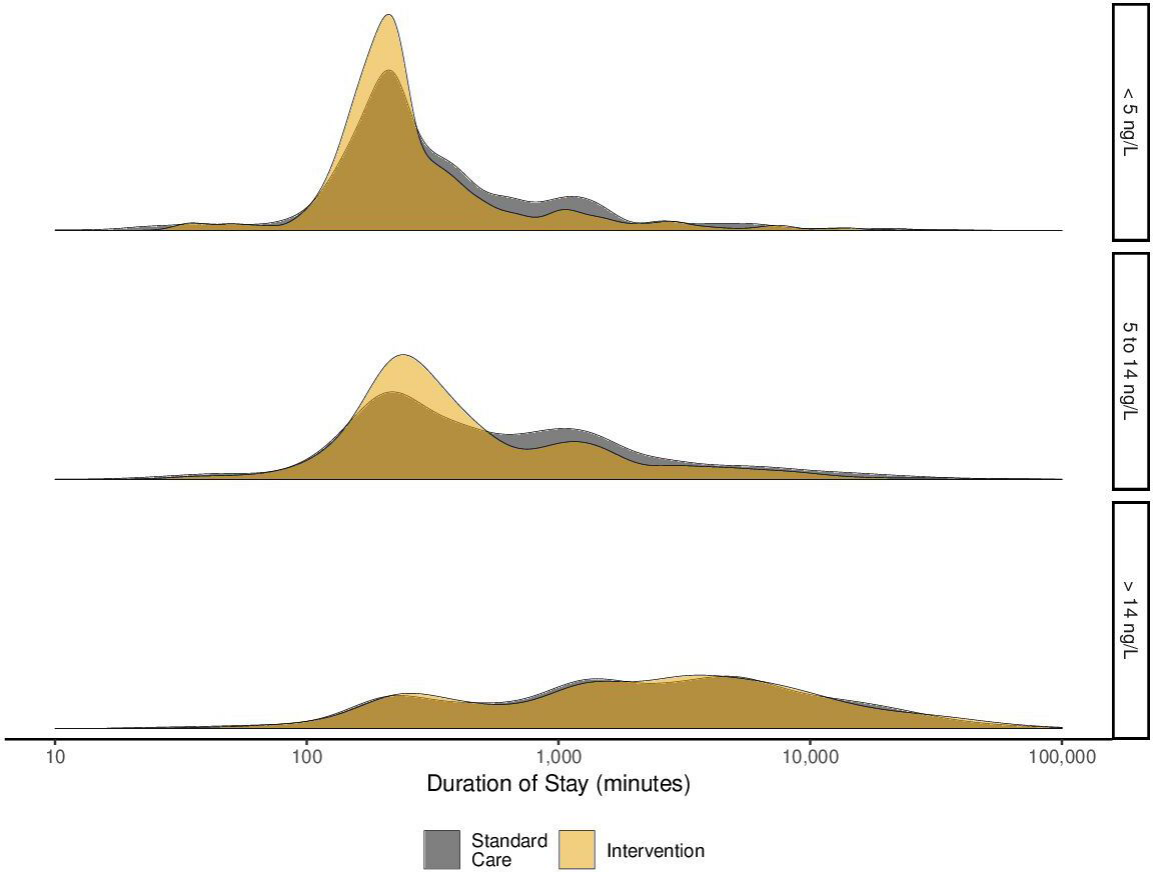
Duration of stay. Density plot illustrating the duration of hospital stay for the standard care (grey) and intervention (yellow) groups stratified according to presentation high-sensitivity cardiac troponin T concentration.

In the overall study population, the primary efficacy endpoint of duration of stay was lower after implementation of the early rule-out pathway (534 (IQR, 220–2279) vs 390 (IQR, 218–1910) min, p<0.001; table 1). In patients with hs-cTnT concentrations below 5 ng/L at presentation, the duration of stay was reduced from 236 (IQR, 180–484) min to 219 (IQR, 170–328) min (p<0.001, figure 2) following implementation of the early rule-out pathway. The reduction in duration of stay from the start to the end of intervention was consistent over time (online supplemental figure 1). In patients with hs-cTnT concentrations between 5 and 14 ng/L, the duration of stay was also reduced following implementation (421 (IQR, 215–1214) min vs 311 (IQR, 215–838) min, p<0.001). The reduction in duration of stay was seen in all patients with hs-cTnT concentrations below the diagnostic threshold (<14 ng/L) (295 vs 255 min) (online supplemental table 1). However, there was no reduction in duration of stay in those patients with an hs-cTnT concentration >14 ng/L (2259 (IQR, 665–6054) min vs 2567 (IQR, 688–6191) min, p=0.363). This observation remained consistent following adjustment for age, sex and comorbidity (online supplemental table 2).

10.1136/openhrt-2021-001769.supp1 Supplementary data

**Figure 3:**
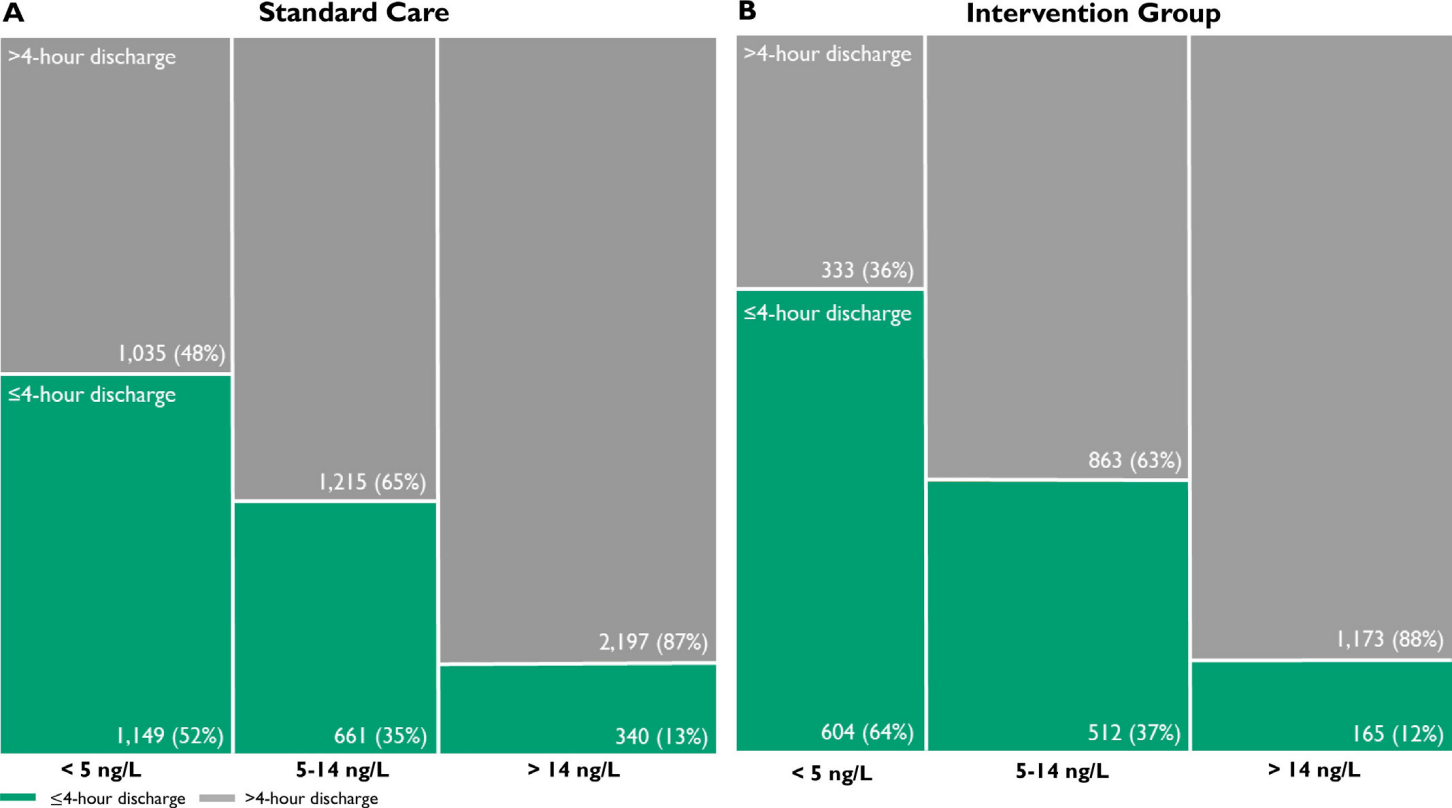
Discharge at 4 hours. Proportion of patients discharged within 4 hours (green) and after 4 hours (grey) before (A) and after (B) implementation of early rule-out pathway stratified according to presentation high-sensitivity cardiac troponin T concentration. There were 68 patients with missing data (45 patients in standard group and 23 in the intervention group).

The secondary efficacy endpoint of the proportion of patients discharged from hospital at 4 hours was higher in the intervention group compared with the standard care group (35.1% vs 32.5%, p=0.011; table 1). The greatest increase was in patients with troponin concentrations <5 ng/L at presentation (63.9% vs 52.5%, p<0.001; figure 3). There was no statistically significant difference in 1-year mortality in those patients <5 ng/L who were discharged <4 or >4 hours from presentation (2 of 604 (0.3%) vs 3 of 341 (0.6%), p=0.953).

### Safety of implementing the early rule-out pathway

**Figure 4:**
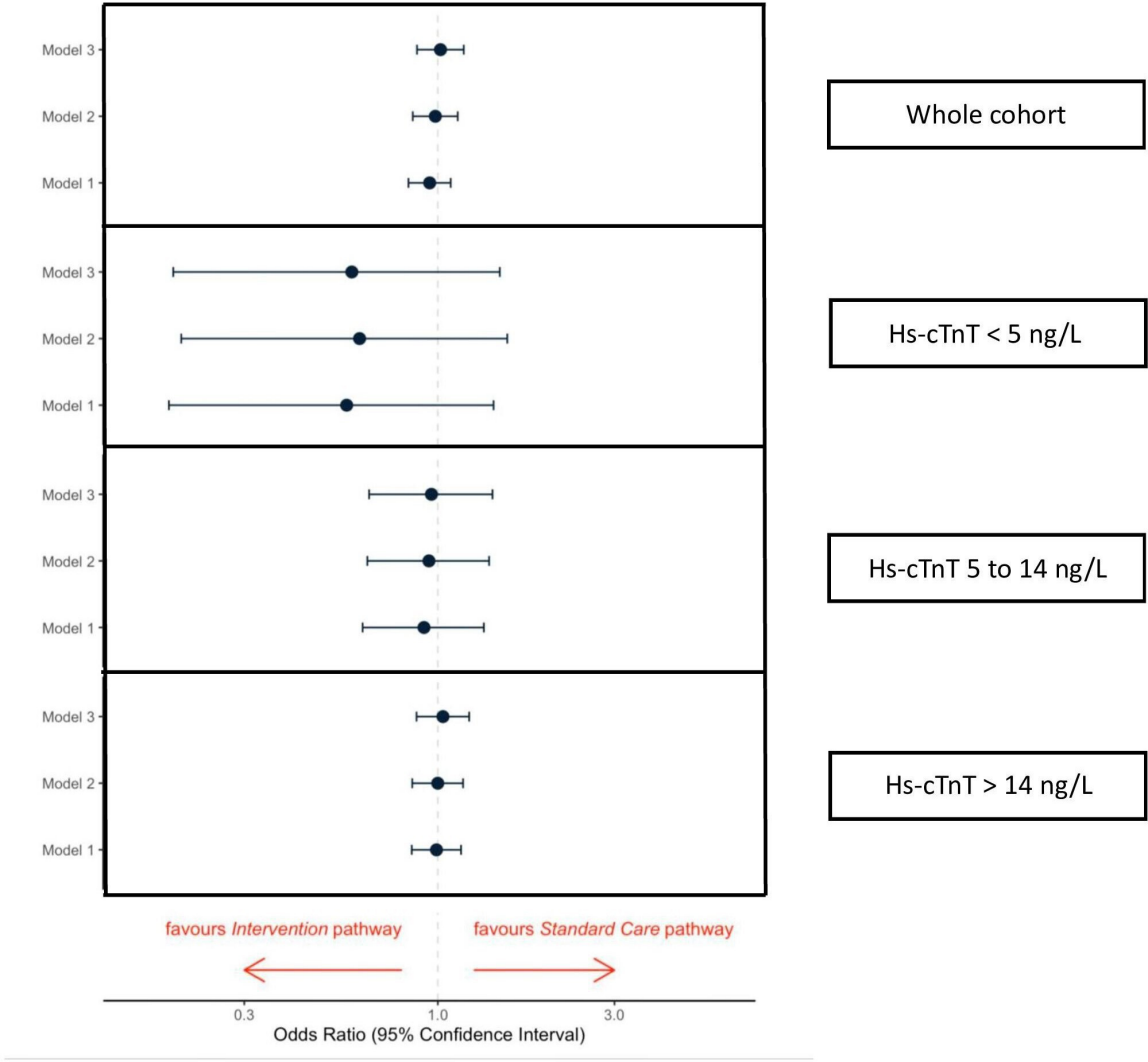
Outcomes. All-cause death at 1 year in all patients admitted following implementation of an early rule-out pathway compared with standard care, and stratified by troponin concentrations at presentation <5 ng/L, between 5 and 14 ng/L, and >14 ng/L. Model 1=crude unadjusted model; model 2=model 1+age+sex; model 3=model 2+sex, creatinine, diabetes mellitus, and a history of myocardial infarction, heart failure or cerebrovascular disease. hs-cTnT, high-sensitivity cardiac troponin T.

A total of 1102 (10.7%) patients died within 1 year (table 1). At 1 year, 10.9% and 10.4% of patients had died before and after implementation of the early rule-out pathway, respectively. Compared with those patients in standard care, there was no statistically significant difference in all-cause death at 1 year following implementation of the early rule-out pathway after adjustment for age, sex, diabetes, creatinine, previous myocardial infarction, heart failure or cerebrovascular disease (OR 1.02, 95% CI 0.88 to 1.18, figure 4). In patients with troponin concentrations <5 ng/L at presentation, 0.8% (17 of 2188) and 0.4% (4 of 945) died before and after implementation of the early rule-out pathway (adjusted OR 0.59, 95% CI 0.19 to 1.47, p=0.291). In patients with troponin concentrations between 5 and 14 ng/L, all-cause death at 1 year occurred in 3.9% and 3.5% before and after implementation of the early rule-out pathways (adjusted OR 0.96, 95% CI 0.65 to 1.41, p=0.839).

Consistent with the primary safety endpoint, there was no statistically significant difference in cardiovascular death at 1 year before and after implementation in all patients with suspected acute coronary syndrome (5.5% vs 5.6%, adjusted OR 1.13, 95% CI 0.93 to 1.36, p=0.221; table 1) or in those with troponin concentrations <5 ng/L (0.2% vs 0.1%, adjusted OR 1.18, 95% CI 0.16 to 6.20, p=0.853), and between 5 and 14 ng/L (1.1% vs 1.2%, adjusted OR 1.19, 95% CI 0.60 to 2.33, p=0.614). We also did not observe a statistically significant difference in all-cause death and cardiovascular death at 30 days between the standard care and intervention groups (online supplemental table 1).

## Discussion

In a large cohort of 10 315 consecutive patients with suspected acute coronary syndrome, we evaluated the effectiveness and safety of implementing an early rule-out pathway using an hs-cTnT concentration of <5 ng/L to risk stratify patients at presentation. Implementation of this approach was associated with a reduction in the duration of hospital stay and an increase in the proportion of patients discharged directly from the emergency department. This was achieved without any change in all-cause or cardiovascular deaths at 1 year, suggesting that the use of this approach to risk stratify patients with suspected acute coronary syndrome is both safe and effective in routine clinical practice.

There are several strengths to our study. First, our before and after study, unlike an individual patient-level randomised controlled trial, enables inclusion of consecutive patients regardless of age, sex, symptoms, time of presentation or comorbidities. Using such an approach limits selection bias and improves the generalisability of our findings. Implementation at the hospital level minimised the risk of a Hawthorn effect from researchers observing care, which may exaggerate the effectiveness of implementing early rule-out pathways. Second, we included a large population of 10 315 patients in the study with over 1000 deaths. We are not aware of any other prospective study of this size evaluating the implementation of an early rule-out pathway using hs-cTnT. Third, given the robust national databases available in Scotland, which have already been used to deliver longitudinal cohort studies and randomised controlled trials, we were able to evaluate the short-term and long-term impact of implementing an early rule-out pathway with all-cause mortality as our primary safety outcome and complete follow-up.^22 24 25^ This approach limits misclassification bias and we further presented cause-specific mortality evaluating cardiovascular deaths.

Our analysis showed that the implementation of an early rule-out pathway led to substantial reductions in the duration of stay in hospital. This observation was likely due to both a change in the threshold for ruling out myocardial infarction and reducing the timing between serial sampling. However, not all patients with an initial troponin concentration <5 ng/L on presentation were discharged according to the pathway. This may be due to ongoing symptoms, early presentations (<3 hours), or other medical and social reasons requiring hospitalisation. Our findings extend the current literature. Using a high-sensitivity troponin I assay, the High-Sensitivity cardiac Troponin On presentation to Rule out myocardial InfarCtion trial demonstrated that implementation of an early rule-out pathway reduced duration of stay by 3.3 hours with no increase in 1-year mortality using a stepped-wedge cluster randomised design in 31 492 patients.^26^ In contrast, the Limit of Detection and ECG Discharge trial showed that implementing an early rule-out pathway using the limit of detection of a range of hs-cTn assays when combined with a normal ECG increased 4-hour discharge rates, but did not significantly reduce duration of stay and the trial was not powered to evaluate safety outcomes.^16^ The Rapid Assessment of Possible acute coronary syndrome In the Emergency Department with high-sensitivity TnT trial compared a 1-hour pathway that incorporates the limit of detection with a 3-hour rule-out pathway using the 99th centile in 3378 patients, and reported that the 1-hour strategy reduced duration of stay by 60 min and increased discharge rates from 32% to 45%.^27^ The trial concluded non-inferiority for a composite primary endpoint that included index events, but there were only 6 deaths at 30 days. However, follow-up at 1 year demonstrated an increase in both subsequent myocardial infarction and death in patients with low troponin concentrations identified by the hs-cTnT assay and randomised to the 1-hour strategy compared with the 3-hour pathway using the 99th centile.^28^ The explanation for these unexpected findings is not clear, but they highlight the importance of conducting adequately powered studies that evaluate both the effectiveness and safety of implementing early rule-out pathways. Furthermore, one other before and after study has evaluated the implementation of an early rule out pathway with hs-cTnT demonstrating a reduction in hospital admissions with no excess in adverse events.^29^ However, this study evaluated the HEART score and a change in hs-cTnT of <3 ng/L to identify low-risk patients.

In our study, implementation of an early rule-out pathway using hs-cTnT reduced duration of stay and was not associated with any change in all-cause or cardiovascular mortality at 1 year. The biggest reduction was seen in the 5–14 ng/L group which likely reflects the change in timing of serial sample from 6 or 12 hours to 3 hours. There was no evidence of harm in models adjusting for age, sex and comorbidity. Studies that have evaluated early rule-out pathways have primarily looked at short-term outcomes with small number of fatal or non-fatal events^16^ or have evaluated hs-cTnI assays.^30^ We assessed safety using both short-term and long-term outcomes. Across the study cohort, we had over 1000 deaths and showed, with a significant degree of confidence, that those patients managed with an early rule-out pathway were not at higher risk overall or when stratified according to hs-cTnT concentration at presentation.

Our pathway was based on the High-STEACS early rule-out pathway^20 31 32^ developed using an hs-cTnI assay. Our study therefore has important clinical implications providing further confidence in early rule-out pathways using a hs-cTnT assay. The findings from our study add to a recently published systematic review of 37 studies evaluating the diagnostic performance of two-step hs-cTn pathways.^33^ This evidence review informed the National Institute for Health and Care Excellence guideline, which recommended pathways using hs-cTn concentrations near the limit of detection at presentation as a first step with a second step of small absolute changes to safely rule out myocardial infarction.^34^ This concept is also recommended by the European Society of Cardiology guidelines using a rule-out threshold of <5 ng/L and a change of <3 ng/L in 0/1-hour or 0/2-hour pathways based on these principles.^35^ Our findings should provide clinicians with additional confidence when adopting early rule-out pathways into clinical practice.

### Study limitations

First, this was a single-centre study in a large secondary care hospital in Scotland, and the impact of adopting this approach may differ in tertiary referral centres or other healthcare settings. Second, the controlled before and after study design is quasi-experimental and less robust than a randomised trial. It is possible that secular trends in practice outside of the pathway could have impacted on duration of stay. However, our stratified analysis demonstrated that the observed reductions in duration of stay were confined to those patients where care would have been modified by the pathway. Nevertheless, we are unable to adjust for unmeasured confounding including changes in clinical practice during the study period which may have contributed to differing clinical characteristics in the standard care and intervention groups. Third, all clinical decisions were made by the attending clinician and therefore not all patients identified as low risk may have been discharged according to our protocol. This may have introduced bias, however our findings are an accurate reflection of clinical practice where judgements are made according to many factors and care pathways are not always followed. Fourth, we did not adjudicate the diagnosis of myocardial infarction or evaluate diagnostic performance. For clinical outcomes, we used routinely collected data. Although this may introduce misclassification, given the same approach was used during both the standard care and implementation phases of our study it is unlikely to have introduced bias. Fifth, while most patients who had an admission cardiac troponin requested would be in the context of suspected acute coronary syndrome, we are unable to exclude patients in whom troponin was requested for other clinical conditions such as pulmonary embolus or acute heart failure. Finally, we used the hs-cTnT thresholds recommended by the manufacturer for use in the UK and the rest of the world, but we are aware the limit of detection varies across different platforms and that the 99th centile recommended for use by the US Food and Drug Administration (USFDA) is higher. Furthermore, the USFDA does not permit the reporting of concentration below 6 ng/L. While the threshold of 5 ng/L used here is likely to have similar effectiveness and safety as using other Roche platforms, we cannot directly inform the safety of implementing this approach using the USFDA thresholds.

## Conclusion

In patients with suspected acute coronary syndrome, implementing an early rule-out pathway, using an hs-cTnT concentration of <5 ng/L at presentation for risk stratification, reduced the duration of stay in hospital without compromising safety. The adoption of this approach in clinical practice could have major benefits for patients and healthcare providers.

## Data Availability

Data are available upon reasonable request. All data relevant to the study are included in the article or uploaded as supplemental information. This study makes use of multiple routine electronic healthcare data sources that are linked, de-identified and held in our safe haven, which are accessible by approved individuals who have undertaken the necessary governance training. All data relevant to the study are included in the manuscript. Summary data can be made available upon request to the corresponding author.
