# Supplementary material for: Implementation of an early rule-out pathway for myocardial infarction using a high-sensitivity cardiac troponin T assay
Source: Open Heart. 2021 Nov 25;8(2):e001769. doi: 10.1136/openhrt-2021-001769 (PMC8627412; doi:10.1136/openhrt-2021-001769)
Supplement: Supplementary data [file openhrt-2021-001769supp001.pdf]

SUPPLEMENTAL APPENDIX

Implementation of an early rule-out pathway for myocardial infarction using a high-sensitivity cardiac troponin T assay

Dennis Sandeman, MSc[a], Maaz BJ Syed, MBChB, MSc[b], Dorien M Kimenai, PhD[c], Kuan K Lee, MD[b], Atul Anand, MD, PhD[b], Shruti Joshi, MD[b], Lorraine Dinnel[a], Philip R Wenham, PhD, MCB, FRCPath[d], Ken Campbell, FIBMS[d], Mary Jarvie BSc[d], Donna Galloway, MPhil[d], Mhairi Anderson, BSc[d], Bappa Roy, MB ChB FRCEM[e], Jack PM Andrews, MD[b], Fiona E Strachan, PhD[b], Amy V Ferry, PhD[b], Andrew R Chapman, MD, PhD[b], Sarah Elsby, BSc[f], Mark Francis, MD[a], Robert Cargill, MD[a], Anoop SV Shah, MD, PhD[g], Nicholas L Mills, MD, PhD[b,c]

[a]Department of Cardiology, Victoria Hospital Kirkcaldy, Kirkcaldy, United Kingdom.
[b]BHF Centre for Cardiovascular Science, University of Edinburgh, Edinburgh, United Kingdom.
[c]Usher Institute, University of Edinburgh, Edinburgh, United Kingdom.
[d]Department of Clinical Biochemistry, Victoria Hospital, Kirkcaldy, United Kingdom.
[e]Emergency Department, Victoria Hospital, Kirkcaldy, United Kingdom.
[f]Infomation Services Department, Lynebank Hospital, Dunfermline, United Kingdom.
[g]Non Communicable Disease Epidemiology, London School of Hygiene and Tropical Medicine, United Kingdom

Address for correspondence:

Dennis Sandeman
Cardiology Department
Victoria Hospital
Kirkcaldy
United Kingdom
KY52AH

Telephone: +44 1595 643355
Email: dennis.sandeman@nhs.scot

Sandeman D, *et al. Open Heart* 2021; 8:e001769. doi: 10.1136/openhrt-2021-001769

Table and figure legends

eTables:

eTable 1. Efficacy and safety endpoints stratified by phase and cardiac troponin concentration

eTable 2. Duration of stay comparing the intervention group to the standard care adjusted for age, sex and comorbidity. Data are provided for the whole population and stratified by troponin concentrations

eFigures:

eFigure 1. Median length of stay before and after the implementation of the new pathway in individuals with hs-cTnT <5 ng/L and stratified into 3-month periods.

eTable 1. Efficacy and safety endpoints stratified by phase and cardiac troponin concentration

	**Standard care**			**Intervention**		
	**Whole Cohort** (n = 6,642)	**Troponin T <14 ng/L on arrival** (n = 4,073)	**Troponin T ≥ 14 ng/L on arrival** (n = 2,569)	**Whole Cohort** (n = 3,673)	**Troponin T <14 ng/L on arrival** (n = 2,325)	**Troponin T ≥ 14 ng/L on arrival** (n = 1,348)
**Duration of hospital stay (minutes)**	534 [220-2279]	295 [192-843]	2259 [665-6054]	390 [218-1910]	255 [190-509]	2567 [688-6191]
**All-cause death**						
30 days	245 (3.7%)	15 (0.4%)	230 (9.0%)	141 (3.8%)	13 (0.6%)	128 (9.5%)
1 year	721 (10.9%)	90 (2.2%)	631 (24.6%)	381 (10.4%)	52 (2.2%)	329 (24.4%)
**Cardiovascular death**						
30 days	139 (2.1%)	5 (0.1%)	134 (5.2%)	82 (2.2%)	9 (0.4%)	73 (5.4%)
1 year	364 (5.5%)	24 (0.6%)	340 (13.3%)	204 (5.6%)	18 (0.8%)	186 (13.8%)

Sandeman D, *et al. Open Heart* 2021; 8:e001769. doi: 10.1136/openhrt-2021-001769

eTable 2. Association of early rule-out pathway with duration of hospital stay

	Beta	(95% Confidence Interval				
**Whole cohort**						
Early rule-out pathway	-0.0135	(	-0.0244	-	-0.0027	)
Age	0.0062	(	0.0059	-	0.0065	)
Sex (men)	0.0025	(	-0.0081	-	0.0132	)
History of myocardial infarction	-0.0131	(	-0.0346	-	0.0085	)
History of heart failure	0.0875	(	0.0606	-	0.1144	)
History of cerebrovascular disease	0.0590	(	0.0160	-	0.1020	)
Diabetes Mellitus	0.0454	(	0.0315	-	0.0592	)
Serum creatinine	0.0007	(	0.0006	-	0.0008	)
**Troponin T below 5 ng/L**						
Early rule-out pathway	-0.0304	(	-0.0442	-	-0.0166	)
Age	0.0019	(	0.0013	-	0.0024	)
Sex (men)	-0.0132	(	-0.0278	-	0.0014	)
History of myocardial infarction	0.0273	(	-0.0122	-	0.0668	)
History of heart failure	0.0865	(	-0.0247	-	0.1976	)
History of cerebrovascular disease	0.2228	(	0.1066	-	0.3390	)
Diabetes Mellitus	0.0587	(	0.0345	-	0.0829	)
Serum creatinine	0.0003	(	-0.0002	-	0.0007	)
**Troponin T between 5 and 14 ng/L**						
Early rule-out pathway	-0.0368	(	-0.0545	-	-0.0192	)
Age	0.0024	(	0.0016	-	0.0031	)
Sex (men)	-0.0236	(	-0.0427	-	-0.0046	)
History of myocardial infarction	-0.0367	(	-0.0710	-	-0.0024	)
History of heart failure	-0.0037	(	-0.0704	-	0.0629	)
History of cerebrovascular disease	0.0252	(	-0.0519	-	0.1022	)
Diabetes Mellitus	0.0170	(	-0.0057	-	0.0397	)
Serum creatinine	0.0004	(	0.0000	-	0.0008	)
**Troponin T over 14 ng/L**						
Early rule-out pathway	0.0099	(	-0.0095	-	0.0294	)
Age	0.0022	(	0.0015	-	0.0029	)
Sex (men)	-0.0381	(	-0.0573	-	-0.0188	)
History of myocardial infarction	-0.0202	(	-0.0526	-	0.0122	)
History of heart failure	0.0445	(	0.0119	-	0.0771	)
History of cerebrovascular disease	0.0145	(	-0.0427	-	0.0716	)
Diabetes Mellitus	0.0064	(	-0.0147	-	0.0275	)
Serum creatinine	0.0003	(	0.0001	-	0.0004	)

Data are presented as β (95% CI) The table presents the association of log-transformed duration of stay for intervention group as compared with standard care for the total study population, and stratified by patients with troponin levels below 5 ng/L, those who had troponin levels between 5 and 14 ng/L, and those who had troponin levels above 14 ng/L. The model is adjusted by age, sex, diabetes, creatinine, and history of myocardial infarction, heart failure or cerebrovascular disease.

Sandeman D, *et al. Open Heart* 2021; 8:e001769. doi: 10.1136/openhrt-2021-001769

BMJ Publishing Group Limited (BMJ) disclaims all liability and responsibility arising from any reliance placed on this supplemental material which has been supplied by the author(s)

eFigure 1

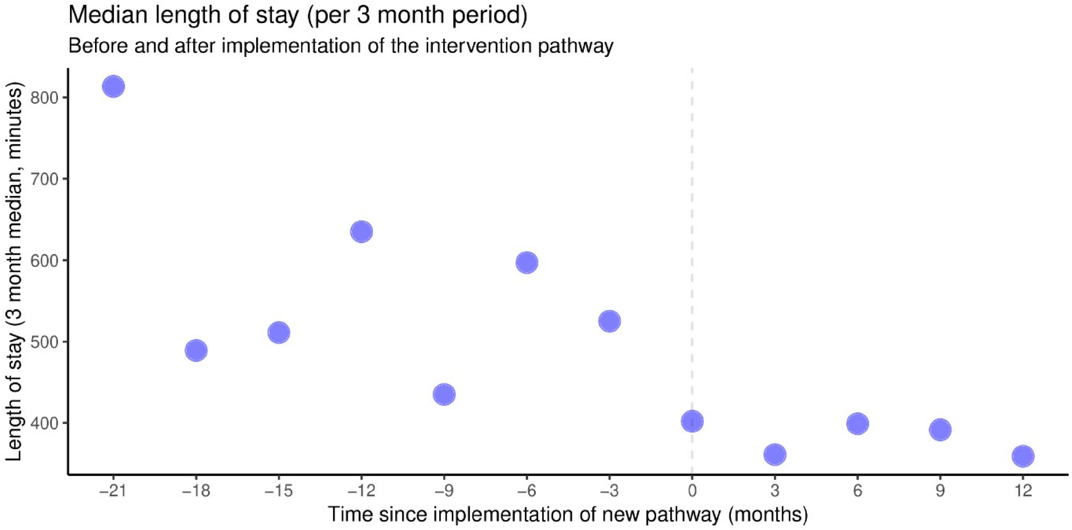

Sandeman D, *et al. Open Heart* 2021; 8:e001769. doi: 10.1136/openhrt-2021-001769
